# Revealing the role of regulatory T cells in the tumor microenvironment of lung adenocarcinoma: a novel prognostic and immunotherapeutic signature

**DOI:** 10.3389/fimmu.2023.1244144

**Published:** 2023-08-21

**Authors:** Pengpeng Zhang, Xiao Zhang, Yanan Cui, Zetian Gong, Wei Wang, Shengrong Lin

**Affiliations:** ^1^ Department of Lung Cancer Surgery, Tianjin Medical University Cancer Institute and Hospital, Tianjin, China; ^2^ Department of Thoracic Surgery, The First Affiliated Hospital of Nanjing Medical University, Nanjing, China; ^3^ Department of Oncology, The First Affiliated Hospital of Nanjing Medical University, Nanjing, China; ^4^ Department of Thoracic Surgery, Dongtai People’s Hospital, Dongtai, China

**Keywords:** lung adenocarcinoma, regulatory T cells, TME, immunotherapy, prognosis

## Abstract

**Background:**

Regulatory T cells (Tregs), are a key class of cell types in the immune system. In the tumor microenvironment (TME), the presence of Tregs has important implications for immune response and tumor development. Relatively little is known about the role of Tregs in lung adenocarcinoma (LUAD).

**Methods:**

Tregs were identified using but single-cell RNA sequencing (scRNA-seq) analysis and interactions between Tregs and other cells in the TME were investigated. Next, we used multiple bulk RNA-seq datasets to construct risk models based on marker genes of Tregs and explored differences in prognosis, mutational landscape, immune cell infiltration and immunotherapy between high- and low-risk groups, and finally, qRT-PCR and cell function experiments were performed to validate the model genes.

**Results:**

The cellchat analysis showed that MIF-(CD74+CXCR4) pairs play a key role in the interaction of Tregs with other cell subpopulations, and the Tregs-associated signatures (TRAS) could well classify multiple LUAD cohorts into high- and low-risk groups. Immunotherapy may offer greater potential benefits to the low-risk group, as indicated by their superior survival, increased infiltration of immune cells, and heightened expression of immune checkpoints. Finally, the experiment verified that the model genes LTB and PTTG1 were relatively highly expressed in cancer tissues, while PTPRC was relatively highly expressed in paracancerous tissues. Colony Formation assay confirmed that knockdown of PTTG1 reduced the proliferation ability of LUAD cells

**Conclusion:**

TRAS were constructed using scRNA-seq and bulk RNA-seq to distinguish patient risk subgroups, which may provide assistance in the clinical management of LUAD patients.

## 1 Introduction

With a significant global incidence and continuing to be a prominent contributor to cancer-related deaths, lung cancer (LC) encompasses two major pathological classifications: small cell lung cancer (SCLC) and non-small cell lung cancer (NSCLC), with NSCLC being the prevailing type. Within the NSCLC category, lung adenocarcinoma (LUAD) stands out as the primary histological subtype, constituting roughly 40% of LC cases (1). In the past decade, significant attention has been focused on early surgical intervention for LC, leading to improved survival outcomes for patients with early-stage disease. Despite advancements in our understanding of LC, the invasiveness and associated risks of LUAD continue to impact patient survival and prognosis. In recent years, targeted therapies utilizing biomarkers have shown promising results in improving survival outcomes for LC patients (2). However, the widespread implementation of this treatment modality requires further exploration. Therefore, exploring additional insights related to LUAD and identifying novel factors associated with patient survival prognosis are crucial for enhancing patient outcomes.

The tumor microenvironment (TME) plays a crucial role in determining tumor development, progression, and patient prognosis (3–5). Tumor growth relies on the presence of a permissive TME, formed through interactions between tumor cells and the stroma, composed of immune cells, fibroblasts, endothelial cells, and extracellular matrix (6). Several studies have described immune characteristics of tumor stroma and defined immune cell subpopulations associated with the prognosis of NSCLC (7–9). Regulatory T cells (Tregs), characterized by the expression of Foxp3 and CD25, have emerged as key players in shaping the immune landscape of the TME. Through their immunosuppressive properties, Tregs impact various aspects of anti-tumor immune responses. They possess the ability to control various types of inflammatory responses by modulating the activity of different cells in both the innate and adaptive immune systems. This wide-ranging control over immunity and inflammation is achieved through the diverse molecular and cellular targets that Tregs interact with (10).

Single-cell RNA sequencing (scRNA-seq) is a high-throughput genomic technology that enables sequencing of the entire genome or transcriptome of individual cells, providing genetic information for each cell (11, 12). With advancements in high-throughput sequencing, scRNA-seq has become a crucial tool in studying cellular heterogeneity and development in the life sciences. Its advantages lie in identifying potential cell subpopulations within a cell population and determining specific gene expression patterns for each subpopulation. Furthermore, it helps elucidate key cellular processes such as cell differentiation, cell cycle, and apoptosis, thereby driving progress in biomedical research and clinical diagnostics. Due to its ability to identify cellular heterogeneity at the single-cell level and avoid the averaging effects of traditional sequencing methods, scRNA-seq has garnered significant attention in fields like oncology, neuroscience, developmental biology, and immunology. The emergence of scRNA-seq has made personalized treatment for cancer patients possible, serving as an effective approach to study tumor heterogeneity and explore underlying mechanisms.

Therefore, we employed scRNA-seq and bulk RNA-seq analysis to detect TME features in LUAD samples, exploring the role of Tregs in the TME. A Tregs-associated score (TRAS) was developed in order to predict the prognosis and response to immunotherapy in patients with LUAD. The findings of our study offer fresh perspectives on the involvement of Tregs in LUAD, thus aiding in the advancement of prognostic biomarkers and the identification of novel molecular therapeutic targets for the treatment of LUAD.

## 2 Methods

### 2.1 Dataset source

The Cancer Genome Atlas (TCGA) database (https://portal.gdc.cancer.gov/) was utilized to obtain bulk RNA-seq data, mutation data, and clinical characteristics of patients diagnosed with LUAD. For the scRNA-seq dataset GSE131907 (13), tissues from 20 LUAD patients, including 11 surgically resected tumor tissue samples, four puncture biopsy samples, and five pleural effusions, were acquired from the Gene Expression Omnibus (GEO) database (http://www.ncbi.nlm.nih.gov/geo/). Additionally, external validation cohorts (GSE13213, GSE26939, GSE29016, GSE30219, and GSE42127) were collected from the GEO database. To ensure data comparability, the expression data was converted to the transcripts per million (TPM) formats. Addressing any batch effects was performed using the “combat” function of the “sva” R package (14). Furthermore, the TCGA database provided bulk sequencing data, mutation data, and clinical details of LUAD patients, which underwent a log2 transformation for standardized data format prior to analysis.

### 2.2 scRNA-seq data analysis

The cell clustering and dimension reduction steps were conducted using the R package “Seurat” (15). Cells were excluded if they exhibited an expression of more than 7,000 or fewer than 300 genes, or if the proportion of unique molecular identifiers (UMIs) derived from the mitochondrial genome exceeded 10%. The dataset’s dimensionality was reduced by applying principal component analysis (PCA) to the variably expressed genes. Subsequently, cluster analysis was performed using the “FindClusters” function, using the top 20 PCA components, and after repeated adjustments, a resolution of 0.8 was selected to better distinguish subgroups. The resulting two-dimensional representation of cell clusters was then annotated using canonical marker genes to identify known biological cell types. To determine the marker genes associated with cell clusters, the Seurat “FindAllMarkers” function was utilized to compare cells within a specific cluster to cells in all other clusters. The “cellchat” R package (16) was used to infer communication networks between cell subpopulations.

### 2.3 Building a high-performance TRAS

Prognostic key genes were identified by conducting univariate Cox regression and lasso regression analyses. Subsequently, a refinement process was performed to select the genes and determine their corresponding coefficients using multivariate Cox regression. The risk score for LUAD patients was calculated using the following formula: Risk score = $\sum_{k=1}^{n}\text{Coef}\left(\text{k}\right)\;\times \text{ Expr}\left(\text{k}\right)$ . Coef (k) represents the abbreviation for regression coefficients, and Expr (k) denotes the expression level of prognostic model genes (17). The risk score calculation was applied to LUAD patients in the dataset, stratifying them into high- and low-risk groups based on the median risk score. The predictive performance of the model was evaluated using receiver operating characteristic (ROC) curves (18, 19), with exceptional performance indicated by area under the curve (AUC) values exceeding 0.65. PCA analysis was employed to visually illustrate the distribution of patients across different risk groups (20, 21).

### 2.4 Nomogram construction

By amalgamating the risk score and clinical characteristics, an enhanced and more precise nomogram was devised utilizing the ‘‘rms’’ R package (22–24), thereby augmenting the prognostic predictive prowess. Stratified analyses grounded on age, pathological T, N, and clinical stage were conducted to evaluate the predictive significance of both the risk score and clinical features.

### 2.5 Enrichment analysis

Gene set variation analysis (GSVA) and gene set enrichment analysis (GSEA) were employed to evaluate the biological characteristics (25, 26). The ssGSEA approach was employed to quantify the enrichment scores of 29 immune signatures (27, 28).

### 2.6 Mutation analysis

The somatic mutations present in the high- and low-risk cohorts of LUAD were thoroughly examined using the “maftools” R package (29–31). The mutation annotation format (MAF) was generated based on the data obtained from the TCGA database. The tumor mutation burden (TMB) of each patient with LUAD was assessed. Visual representation of the mutation landscape and immune infiltration scores was achieved using the “ComplexHeatmap” R package (32). TCGA-LUAD patients were stratified into four distinct groups based on median risk score and median TMB, and their survival disparities were compared based on the median risk score and TMB.

### 2.7 The TME and immunotherapy

Seven immune infiltration algorithms were utilized to evaluate the immune cell content by accessing the timer 2.0 database (http://timer.comp-genomics.org/). Heatmaps were employed to visually represent the variances in immune cell infiltration across different risk groups. Additionally, the immunological scores, stromal scores, and ESTIMATE scores of LUAD patients were calculated using the “estimate” R package (33). To forecast the responsiveness to immunotherapy, the Cancer Immunome Atlas (TCIA) database was explored for Immunophenoscores (IPS) associated with TCGA-LUAD. A comparison of IPS was carried out between the high-risk and low-risk groups in this study (34). The “oncoPredict” R package was used to predict potentially effective chemotherapeutic agents between the risk groups (35).

### 2.8 Cell lines culture and qRT-PCR

Ethical approval (Approval No. 2019-SR-156) was obtained from the Medical Ethics Committee for tissue specimens acquired from the First Affiliated Hospital of Nanjing Medical University. These specimens were stored at a temperature of -80°C. A total of ten pairs of samples were collected from LUAD patients who underwent tumor resection, including tumor tissue (T) and precancerous tissue (N). Normal human lung epithelial cells, known as BEAS-2B cells, as well as human LUAD cell lines represented by A549 and H1299 cells, were procured from the Cell Resource Center of Shanghai Life Sciences Institute. The cells were cultured in F12K or RPMI-1640, supplemented with 10% fetal bovine serum (FBS), 1% streptomycin, and penicillin. Maintained under conditions of 37°C, 5% CO2, and 95% humidity, the cell cultures were established (26, 36, 37). Total RNA from LUAD cells or tissues was isolated using the TRIzol reagent by Thermo Fisher Scientific, based in Waltham, MA, USA. The cDNA synthesis was carried out according to the manufacturer’s protocol, utilizing the RevertAid™ First Strand cDNA Synthesis Kit provided by Thermo Fisher Scientific. Subsequently, a qRT-PCR assay was performed on a StepOne Real-Time PCR system, also manufactured by Thermo Fisher Scientific, using a SYBR Green PCR kit from Takara Bio in Otsu, Japan (38). The quantification of relative gene expression levels was conducted using the 2^-△△CT^ method.

### 2.9 Colony formation

A transfection was conducted on 1000 cells, which were then placed in 6-well plates for approximately 14 days. After a period of two weeks, the cell clones were visually inspected without magnification. Subsequently, the cells were washed and subjected to fixation in 4% paraformaldehyde (PFA) for 15 minutes. Staining with crystal violet from Solarbio, China, was performed for 20 minutes, followed by air drying at room temperature. The cell count per well was subsequently calculated.

### 2.10 Statistical methods

R, specifically version 4.2.0, was employed for the statistical analyses and data processing procedures. To establish statistical significance, Kaplan-Meier curves were utilized for survival analysis (39), and the log-rank test was employed. The generation of all survival curves was accomplished using the “survminer” R package (40). Heatmaps were created utilizing the “pheatmap” R package. For variables exhibiting a normal distribution, quantitative differences were assessed using either a two-tailed t-test or a one-way analysis of variance (41, 42). In the case of non-normally distributed data, the implementation of the Wilcoxon test or the Kruskal-Wallis test was conducted (41, 43). All statistical analyses were performed within the R environment, considering a p-value threshold of< 0.05 as indicating statistical significance (44).

## 3 Results

### 3.1 The scRNA profiling of LUAD

**Figure 1:**
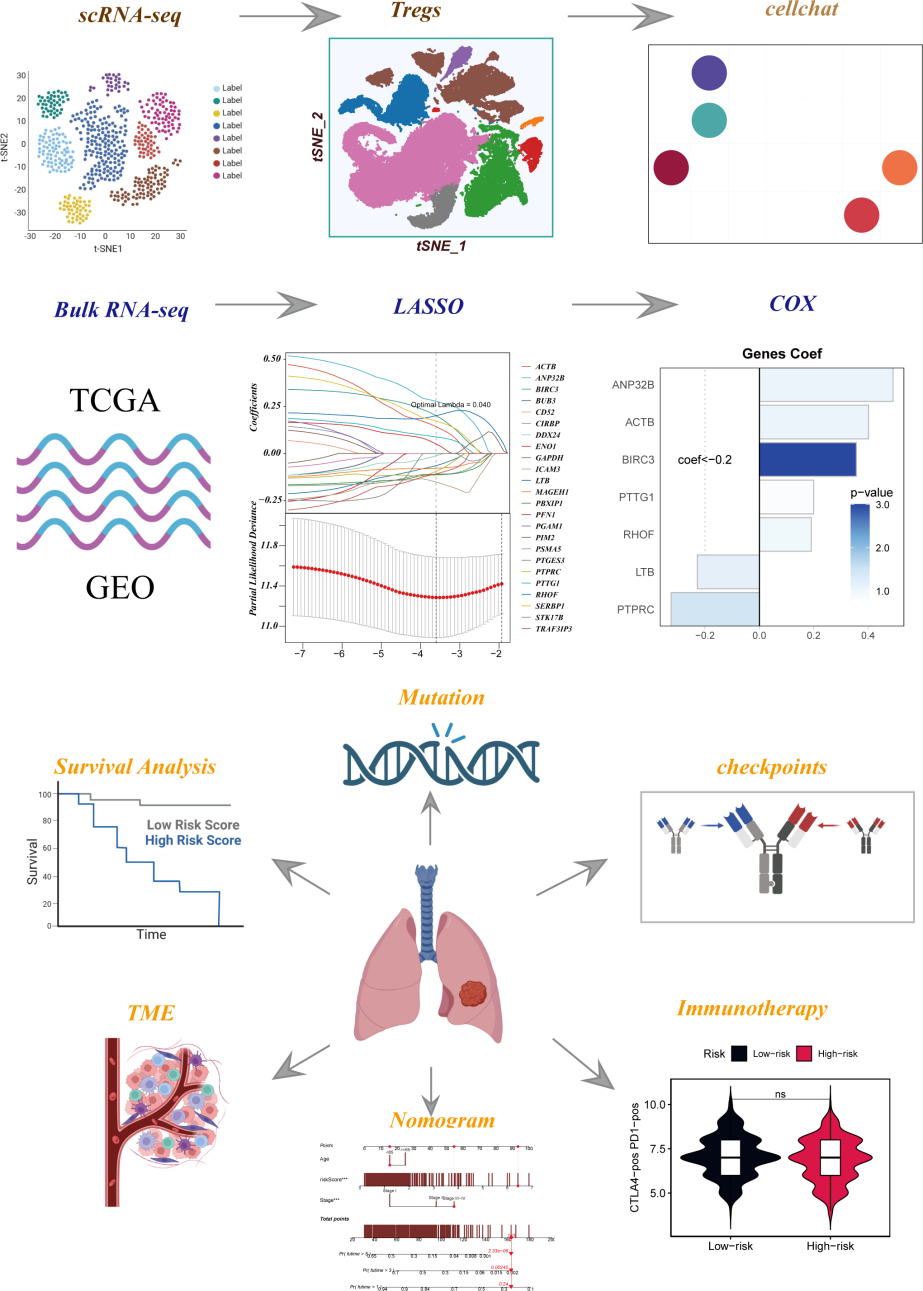
Flow chart of all analyses.

**Figure 2:**
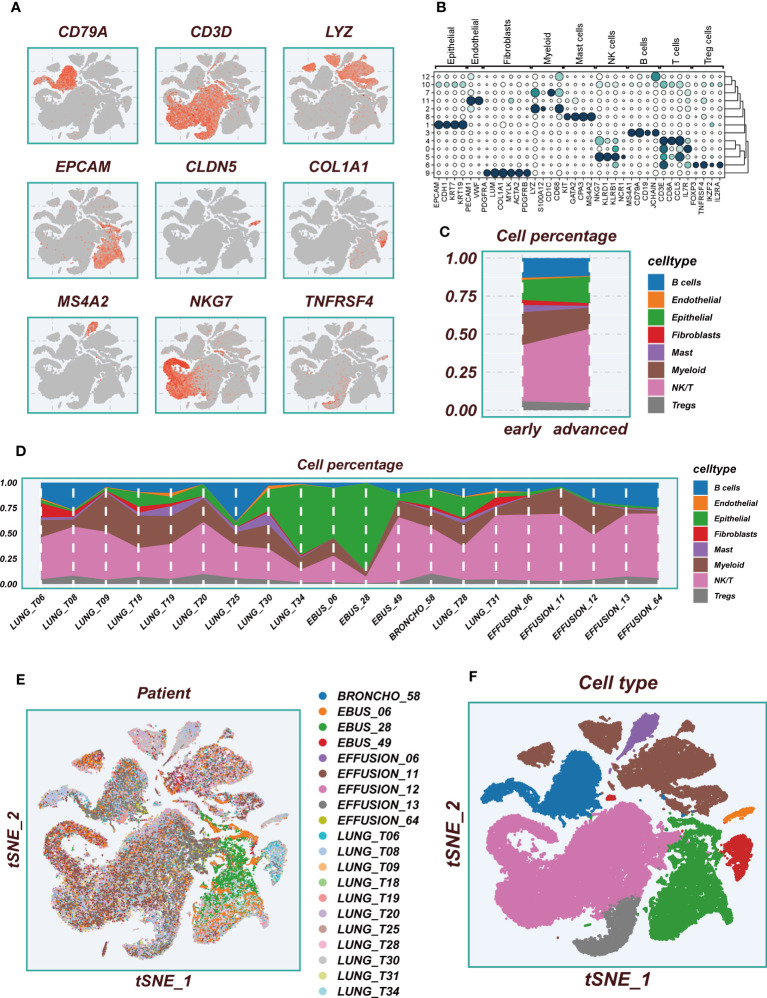
Flow chart of single cell analysis. **(A)** Multiple tSNE plots showing the expression of classic cell type marker genes. **(B)** Bubble plots showing the expression of marker genes corresponding to each cluster. **(C)** A histogram of the percentage of cellular components showing the changes in the proportion of different cellular subpopulations at early and advanced LUAD. **(D)** A histogram showing the variation of cell proportions between different samples. **(E)** A tSNE plot showing the distribution of cell samples from different LUAD tissues. **(F)** A tSNE plot demonstrating the distribution of different cell types.

The flowchart pertaining to the study was presented in **Figure 1**. Following a meticulous evaluation encompassing the proportion of cellular signatures and the expression of mitochondrial and ribosomal genes, a grand total of 73,813 cells displaying exemplary quality were discerned as suitable for subsequent scrutiny. **Supplementary Figures S1A, B** delineate the expression characteristics exhibited by each individual sample. Noteworthy fluctuations in cell cycles were absent from the principal component analysis (PCA) reduction plot as evidenced in **Supplementary Figure S1C**. **Figure 2A** vividly portrays characteristic indicators for distinct cell types. The bubble diagram (**Figure 2B**) visually encapsulates the expression level of the marker gene associated with each cluster. **Figure 2C** showed the percentage of cell types in the early and advanced LUAD samples. **Figure 2D** illustrates the distribution of various cell types as a percentage in the 20 LUAD samples derived from diverse sources. A pervasive uniformity was noted in the cellular distribution within each sample, implying the conspicuous absence of discernible batch effects that could potentially exert an influence on subsequent investigations (**Figure 2E**). Subsequently, the employment of dimensionality reduction methodologies, particularly t-distributed Stochastic Neighbor Embedding (tSNE), culminated in the categorization of all cells into twelve discrete clusters (**Supplementary Figure S1D**). A tSNE plot was used in **Figure 2F** to show the distribution of each cell population. A UMAP figure in **Supplementary Figure S1E** depicted the distribution of each cell group. **Supplementary Figure S1F** displayed t-SNE plots depicting the distribution of cells at various stages of development.

### 3.2 Cell-cell interactions

**Figure 3:**
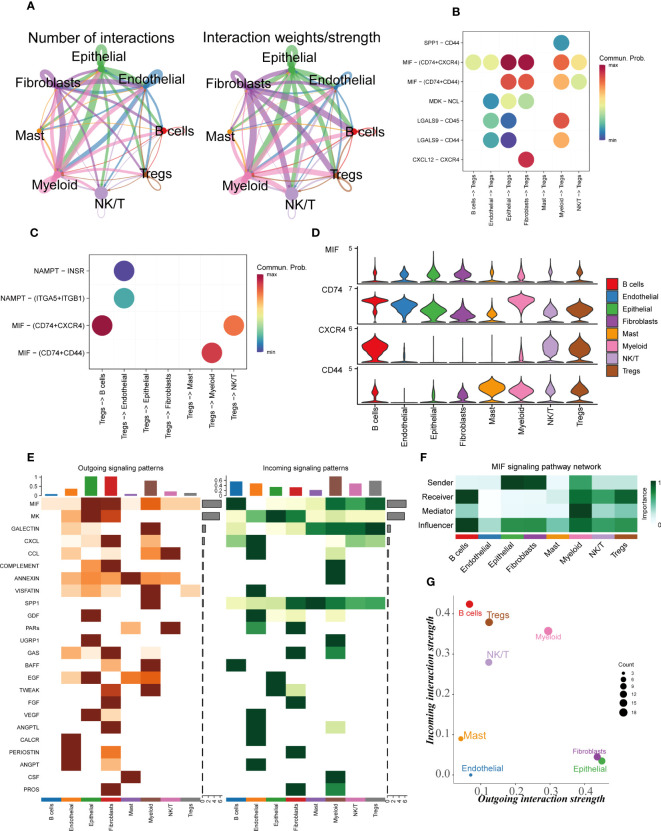
Cellular interactions analysis. **(A)** Showing the number and intensity of interactions between different cell populations in the TME. **(B, C)** Bubble plots showing the possible ligand-receptor pairs between Tregs and other cell subpopulations in the TME. **(D)** Expression of key ligand-receptor pair genes in cell populations. **(E)** Heatmaps demonstrating the strength of outgoing and incoming signaling pathways in different cell subpopulations. **(F)** The roles played by different cell populations in the tumor microenvironment in the MIF signaling pathway network. **(G)** A scatter plot showing the distribution of different cell populations in the intensity of outgoing and incoming signaling interactions.

Within the TME, there are robust cellular interactions involving regulatory T cells (Tregs) and various components, such as epithelial cells, fibroblasts, and myeloid cells (**Figure 3A**). These interactions play a crucial role in shaping the immune response and tumor progression. Epithelial cells and fibroblasts are capable of communicating with Tregs through the interaction of MIF-(CD74+CXCR4) pairs (**Figure 3B**). Similarly, investigations have revealed that Tregs can engage in interactions with B cells via MIF-(CD74+CXCR4) pairs (**Figure 3C**). This suggests that the MIF-(CD74+CXCR4) axis might be a critical signaling pathway in facilitating the crosstalk between Tregs and various cell populations within the TME. The expression levels of CD74, CXCR4, and CD44 genes were found to be significantly upregulated in Tregs (**Figure 3D**). This upregulation may indicate an increased capacity of Tregs to respond to signaling cues from neighboring cells, reinforcing their role as key regulators of the immune response within the TME. Of particular significance, macrophage migration inhibitory factor (MIF) appears to play a pivotal role in mediating these intricate interactions between Tregs and other cell populations within the TME (**Figure 3E**). MIF is known to be involved in modulating immune responses and inflammation, and its involvement in the TME may have far-reaching implications for tumor immune evasion and progression. Notably, Tregs cells seem to predominantly function as receivers in this context, actively receiving signals from various neighboring cells (**Figures 3F, G**). This highlights their ability to sense and respond to the cues provided by other cell types, allowing them to orchestrate immune tolerance and suppress anti-tumor immunity. The significance of these cell-to-cell communications lies in their potential to influence the immune landscape within the TME. By interacting with epithelial cells, fibroblasts, myeloid cells, and B cells, Tregs may contribute to immune suppression, tumor immune evasion, and tumor progression. Understanding these complex interactions could offer new insights into the development of immunotherapeutic strategies aimed at disrupting these suppressive signals and restoring anti-tumor immune responses. GO and KEGG enrichment analysis of Tregs cells marker genes showed that main enriched pathways were the interaction between cytokines and the activation of T cells (**Supplementary Figures S2A, B**).

### 3.2 Constructing TRAS

**Figure 4:**
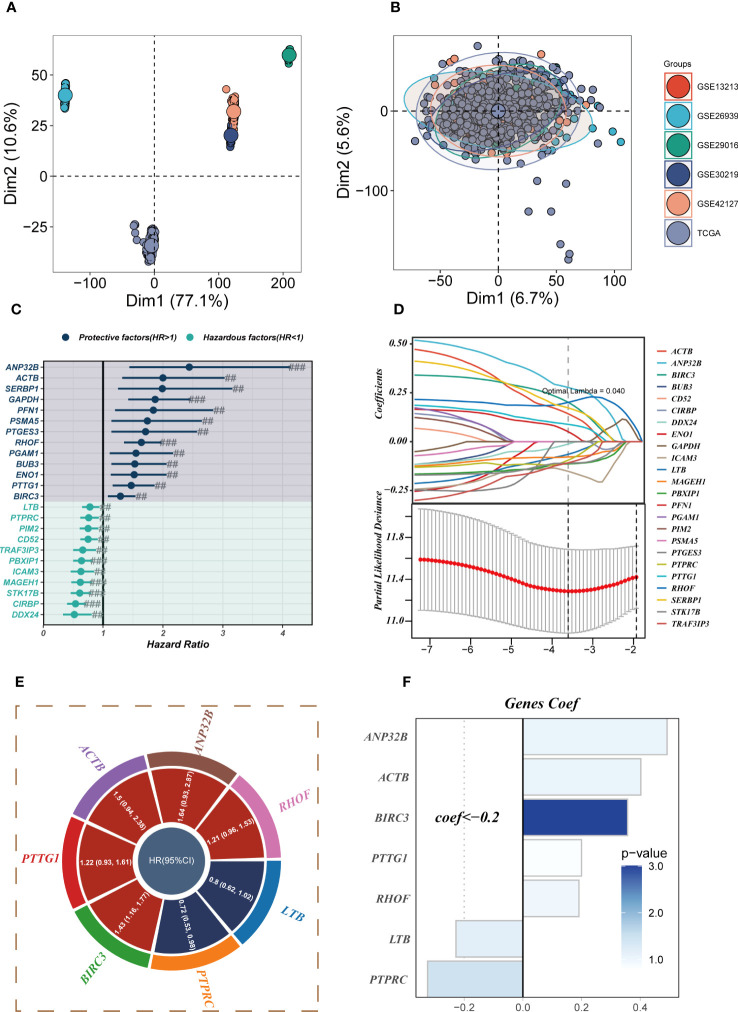
Construction of a stable risk model. **(A, B)** Sample distribution characteristics of multiple bulk RNA-seq cohorts before and after removal of batch effects. **(C)** Forest plot showing the results of univariate COX analysis. **(D)** LASSO regression screening for significant variables affecting prognosis. **(E)** Circle plot showing genes included in the risk model after multivariate regression analysis. **(F)** Distribution of coefficient values of model genes. # represents p<0.05; ## represents p<0.01; ### represents p<0.001.

The six datasets utilized in the analysis were subjected to de-batching procedures to account for any batch effects (**Figures 4A, B**), with the TCGA cohort serving as the reference cohort for the model construction. The marker genes of Tregs cells were intercrossed with bulk sequencing data genes, and a total of 298 genes overlapped (**Supplementary Figure S2C**). Prognostic models incorporating Tregs marker genes were developed through the application of COX and Lasso regression analyses. The methodology employed for identifying essential prognostic variables is illustrated in **Figures 4C, D**. **Figure 4E** provides the hazard ratio (HR) values associated with each variable included in the model, while **Figure 4F** showcases the corresponding coefficients of specific variables. (**Supplementary Figure S2D**) demonstrates that TRAS is an independent prognostic model (*P*<0.001).

### 3.3 Model evaluation

**Figure 5:**
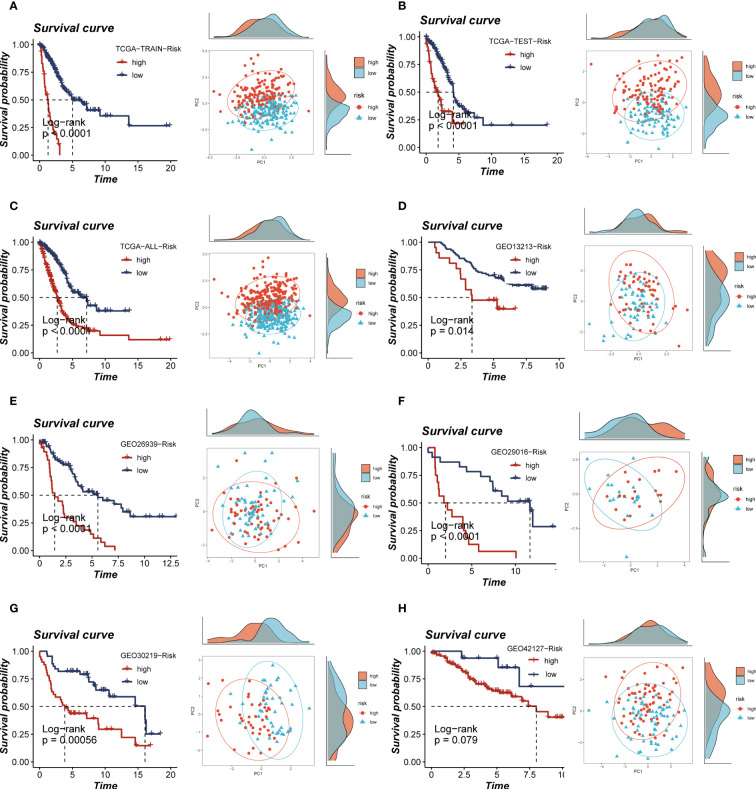
Survival curves and sample distribution of high- and low-risk groups. **(A–H)** The survival differences and PCA sample distribution of different risk groups in TCGA-TRAIN, TCGA-TEST, TCGA-ALL, GEO13213, GEO23939, GEO29016, GEO30219 and GEO42127, respectively, were presented.

The calculation of the risk score for each patient was performed by multiplying the model gene expression with the corresponding coefficients. Subsequently, the patients were categorized into high- and low-groups based on the median value of the risk score. Notably, the high-risk groups of both the TCGA and the five GEO cohorts showed a poor prognosis, and the PCA analysis showed that the high- and low-risk group samples could be clearly divided into two clusters, thus demonstrating the accuracy and stability of the proposed model (**Figures 5A–H**). The ROC curves demonstrate the exceptional predictive ability of TRAS in prognosticating outcomes (**Supplementary Figures S3A–F**). Based on the expression levels of model genes, patients with LUAD were categorized into high- and low-expression groups. Among these groups, patients with elevated expression of LTB and PTPRC genes exhibited a more favorable prognosis, whereas those with heightened expression of BIRC3, PTTG1, ACTB, ANP32B, and RHOF experienced improved survival rates (**Supplementary Figures S4A–G**).

### 3.4 Clinical correlation and nomogram construction

**Figure 6:**
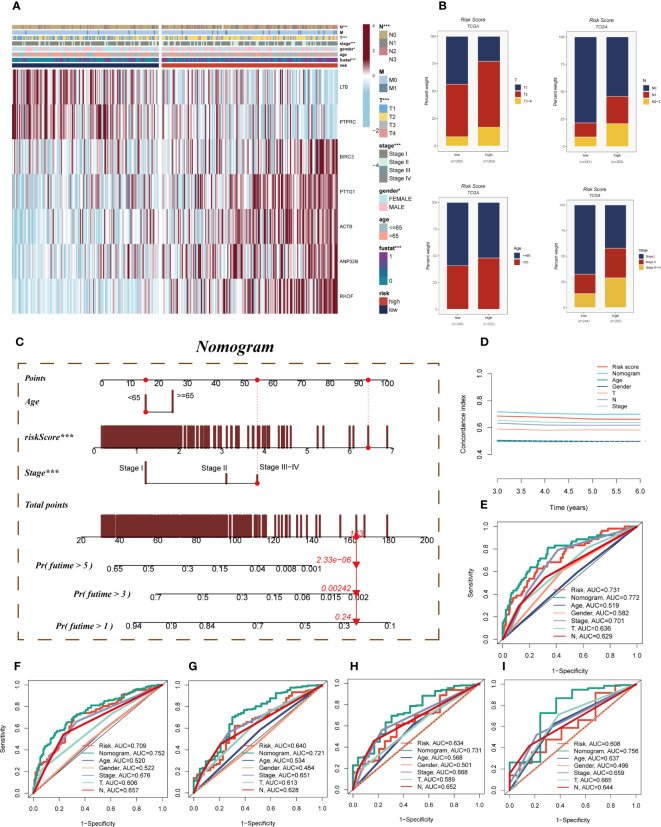
Clinical correlation analysis and construction of the nomogram. **(A)** A heatmap was constructed by combining clinical features and model gene expression to demonstrate the distribution of clinical features and model genes in high- and low-risk groups. **(B)** Bar plots showing the proportion of T-stage, N-stage, age group, and clinical stage in the high- and low-risk groups. **(C)** A nomogram was constructed by combining age, risk score and clinical stage. **(D)** Concordance index curves showing the performance comparison of clinical characteristics, risk scores and nomogram scores. **(E–I)** ROC curves showing AUC values for clinical characteristics, risk scores and nomogram scores at 1-, 3-, 5-, 7-, and 10-years, respectively. Note: ***P < 0.001.

A heatmap was devised to depict the distribution of clinical characteristics among distinct risk groups by amalgamating clinical information and the high- and low-risk subgroups (**Figure 6A**). The high-risk cohort exhibited an association with relatively advanced T-stage, N-stage, and clinical stage, thereby indicating a relatively unfavorable prognosis for patients belonging to this group (**Figure 6B**). To assess the risk of TCGA-LUAD patients, a nomogram was formulated by integrating the clinical features and risk groups. **Figure 6C** visually portrays the stage, age, and risk score of the patients, thereby providing a valuable tool for a more precise risk assessment and guiding future treatment decisions. In terms of performance, the nomogram scores were found to surpass other clinical features and risk scores, as demonstrated by the C-index curves (**Figure 6D**). Both the calibration curve and the decision curves indicated that the Nomogram score had good predictive performance (**Supplementary Figures S5A, B**). Moreover, the prognostic ROC analysis was employed to evaluate the accuracy of the nomogram, revealing significantly superior performance in comparison to other clinical features and risk scores. It is important to note that the AUC values for 1, 3, 5, 7, and 10 years were 0.772, 0.752, 0.721, 0.731, and 0.756, respectively (**Figures 6E–I**).

### 3.5 Mutation landscape

**Figure 7:**
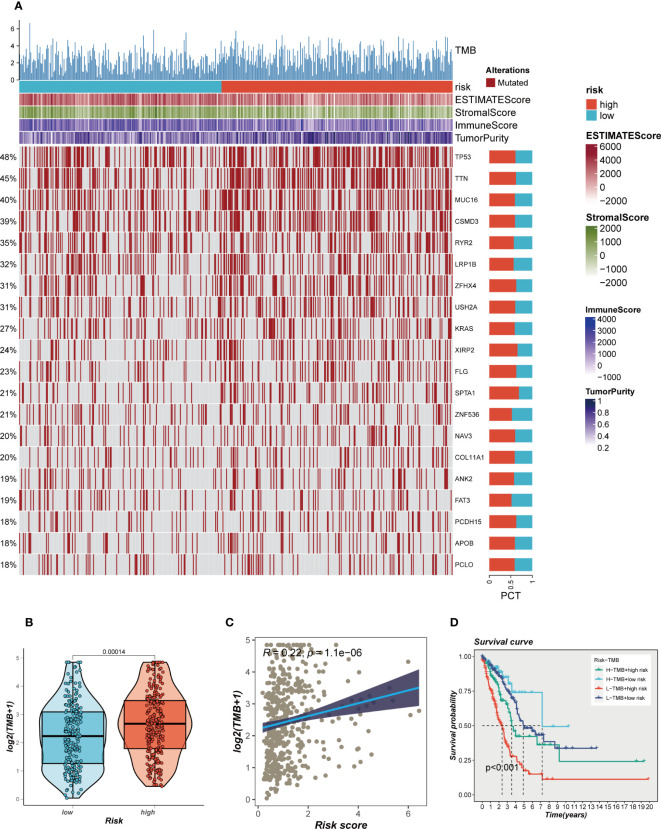
Mutation landscape in high- and low-risk groups. **(A)** Heat map of sample mutation differences between high- and low-risk groups. **(B)** Boxplots of the difference in TMB windiness between high and low risk groups. **(C)** Scatter plot of correlation between risk scores and TMB. **(D)** Survival curves showing the difference between survival among four subgroups (high-risk and high- mutation, high-risk and low-mutation, low-risk and high-mutation, low-risk and low-mutation).

The impact of somatic mutations on the outcomes of cancer immunotherapy exhibits variability. The mutational profile of TCGA-LUAD was scrutinized, and the findings are delineated in **Figure 7A**. Furthermore, a comparative analysis of the disparities in TMB between the high- and low-risk groups revealed relatively augmented mutation rates in the high-risk cohort of LUAD (**Figure 7B**). A significant positive correlation between TMB and risk score was identified through Spearman correlation analysis (R = 0.22, P<0.05, **Figure 7C**). Based on the median values of TMB and risk score, patients were categorized into four groups. The analysis revealed that patients with high-mutation and low-risk LUAD exhibited the most favorable prognosis, while those with low-mutation and high-risk LUAD demonstrated the poorest prognosis (**Figure 7D**).

### 3.6 Enrichment analysis

**Figure 8:**
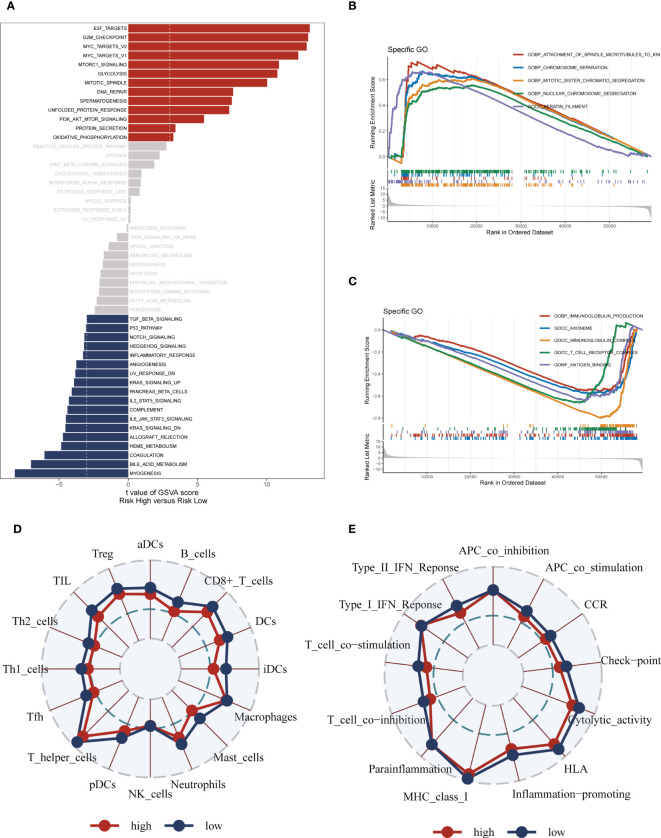
Enrichment pathways between different risk groups. **(A)** GSVA enrichment analysis demonstrates the enrichment of hallmark gene sets between different risk groups. **(B, C)** GSEA enrichment analysis demonstrating the enrichment of differential genes to GO pathways between high- and low-risk groups. **(D, E)** ssGSEA enrichment analysis demonstrating the enrichment of immune cell infiltration and immune-related pathways between high- and low-risk groups.

The GSVA analysis revealed significant pathway enrichments in the high-risk group, including E2F targets, the G2M checkpoint, and mTORC1 signaling (**Figure 8A**). Additionally, the GSEA enrichment analysis visually depicted notable pathway enrichments in the high-risk group, such as the Attachment of spindle microtubules to kinetochores, Chromosome segregation, and Mitotic sister chromatid segregation (**Figures 8B, C**). On the other hand, the low-risk group showed prominent enrichment in the Immunoglobulin production pathway (**Supplementary Figure S5C**). Immunoglobulin production is a vital process through which the immune system generates antibodies, essential proteins produced by B cells to defend the body against pathogens and foreign substances. Furthermore, the ssGSEA analysis indicated that the low-risk group exhibited a higher abundance of infiltrating immune cells, specifically B-cells and antigen-presenting dendritic cells (aDCs) (**Figures 8D, E**). These findings collectively suggest that patients in the high-risk group are characterized by significant alterations in key cellular pathways associated with cell cycle regulation and signaling, while the low-risk group appears to have a heightened immune response indicated by increased levels of infiltrating B-cells and aDCs, and enriched Immunoglobulin production pathway activity.

### 3.7 Immune infiltration assessment

**Figure 9:**
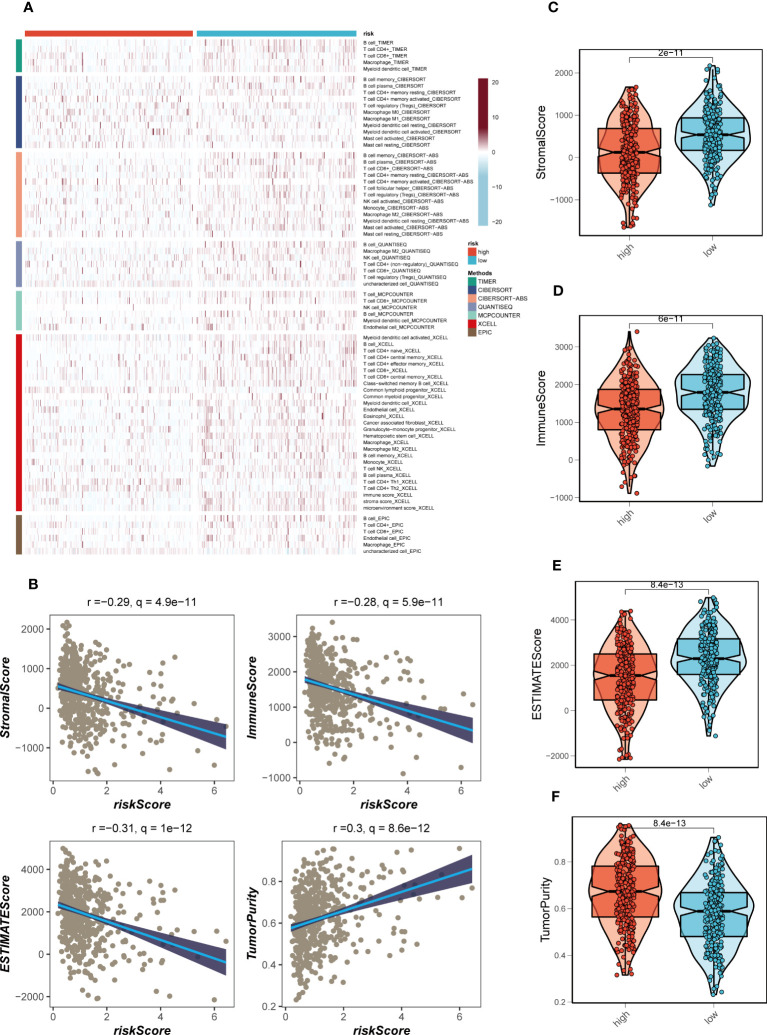
Immune infiltration assessment. **(A)** Heat map demonstrating the differences in immune cell infiltration between high- and low-risk groups assessed using seven algorithms. **(B)** Scatter plot of correlation between risk score and stromal score, immune score, ESTIMATE score, and tumor purity. **(C–F)** Boxplots of differences between risk groups in stromal score, immune score, ESTIMATE score and tumor purity.

The disparities in immune infiltration between the high- and low-risk groups in TCGA-LUAD were assessed by utilizing the data obtained from seven immune infiltration algorithms in the TIMER database. The analysis revealed that the low-risk group exhibited relatively higher levels of immune infiltration (**Figure 9A**). Furthermore, the levels of immune infiltration in the distinct risk groups were validated using the ESTIMATE method, which allows for the estimation of tumor purity and stromal fraction in the TME. The Spearman correlation analysis was performed to investigate the association between the risk score and the immune infiltration score, revealing a significant negative correlation (R = -0.28, FDR< 0.001, **Figure 9B**). This correlation suggests that as the risk score increases, there is a decrease in the extent of immune cell infiltration in the TME. To gain further insights into the immune landscape of the different risk groups, we evaluated the immune scores derived from the ESTIMATE method. Higher immune scores were observed in the low-risk group compared to the high-risk group (P< 0.05, **Figures 9C–F**). The immune score represents the abundance of immune cells within the TME, and the higher immune score in the low-risk group suggests a higher proportion of immune cell infiltration in this group. The association between risk score, immune infiltration, and the immune scores has important implications. The negative correlation between risk score and immune infiltration indicates that the high-risk group, characterized by unfavorable gene expression patterns, is associated with a reduced immune cell presence in the TME. This could potentially create an immunosuppressive microenvironment, hampering anti-tumor immune responses and promoting disease progression in LUAD patients. On the other hand, the low-risk group, which exhibits a more favorable gene expression profile, shows higher immune scores and increased immune cell infiltration in the TME. This suggests a more immune-active microenvironment, which may facilitate anti-tumor immune responses and improve the efficacy of immunotherapy in these patients. Taken together, these findings underscore the association between the risk score, immune infiltration, and the immune microenvironment in LUAD.

### 3.8 Immunotherapy and chemotherapy drugs

**Figure 10:**
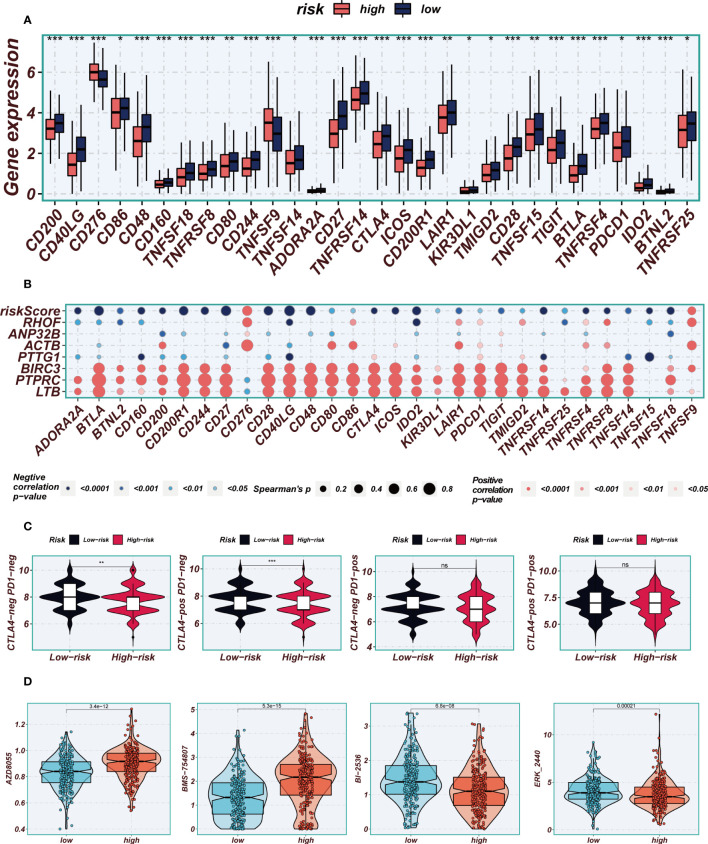
Immune checkpoint and immunotherapy analysis. **(A)** Boxplots showing the difference in immune checkpoint expression between high- and low-risk groups. **(B)** Correlation scatter plots showing the correlation between model genes and risk scores and immune checkpoint expression. **(C)** TCIA analysis showing the difference in IPS scores between different risk groups to infer the possible benefit of receiving PD-1 and CTLA-4 treatment in different risk groups. **(D)** Boxplots demonstrating the possible sensitivity of chemotherapeutic agents between different risk groups. Note: *P < 0.05, **P < 0.01, ***P < 0.001.

The application of immune checkpoint blockade has been widely utilized in advanced LUAD patients. The relationship between well-established immune checkpoints and risk scores in the TCGA-LUAD cohort was analyzed. It was observed that higher expression of almost all immune checkpoint genes (ICGs), such as CD48, CD40LG, and CD27, was found in the low-risk group (**Figure 10A**). Bubble plots were used to visualize the correlation between model genes, risk scores, and ICGs (**Figure 10B**), where blue indicated negative correlation and orange indicated positive correlation. Interestingly, a positive correlation was observed between the expression levels of model genes and most immune checkpoints, while risk scores exhibited a negative correlation with the expression levels of certain common immune checkpoints, including ADRA2A, BTLA, BTNL2, CD160, and CD244. These findings provide valuable insights into the potential of immune checkpoint blockade therapy for LUAD patients. In order to evaluate the potential benefits of immunotherapy among different risk groups, the IPS scores were compared across various risk groups to identify patients who may derive greater advantages from immunotherapy. The tumor samples from these individuals were predicted to exhibit favorable immune responses to PD-L1 or CTLA4 inhibitors, or both (**Figure 10C**). Significantly higher IPS scores were observed in the low-risk group, indicating that they would derive the most benefit from this type of immunotherapy. Drug sensitivity analysis indicates that AZD8055 and BMS-754807 potentially exhibit enhanced efficacy in the low-risk group, whereas BI-2536 and ERK_2440 demonstrate superior sensitivity in the high-risk group (**Figure 10D**).

### 3.9 Experimental validation

**Figure 11:**
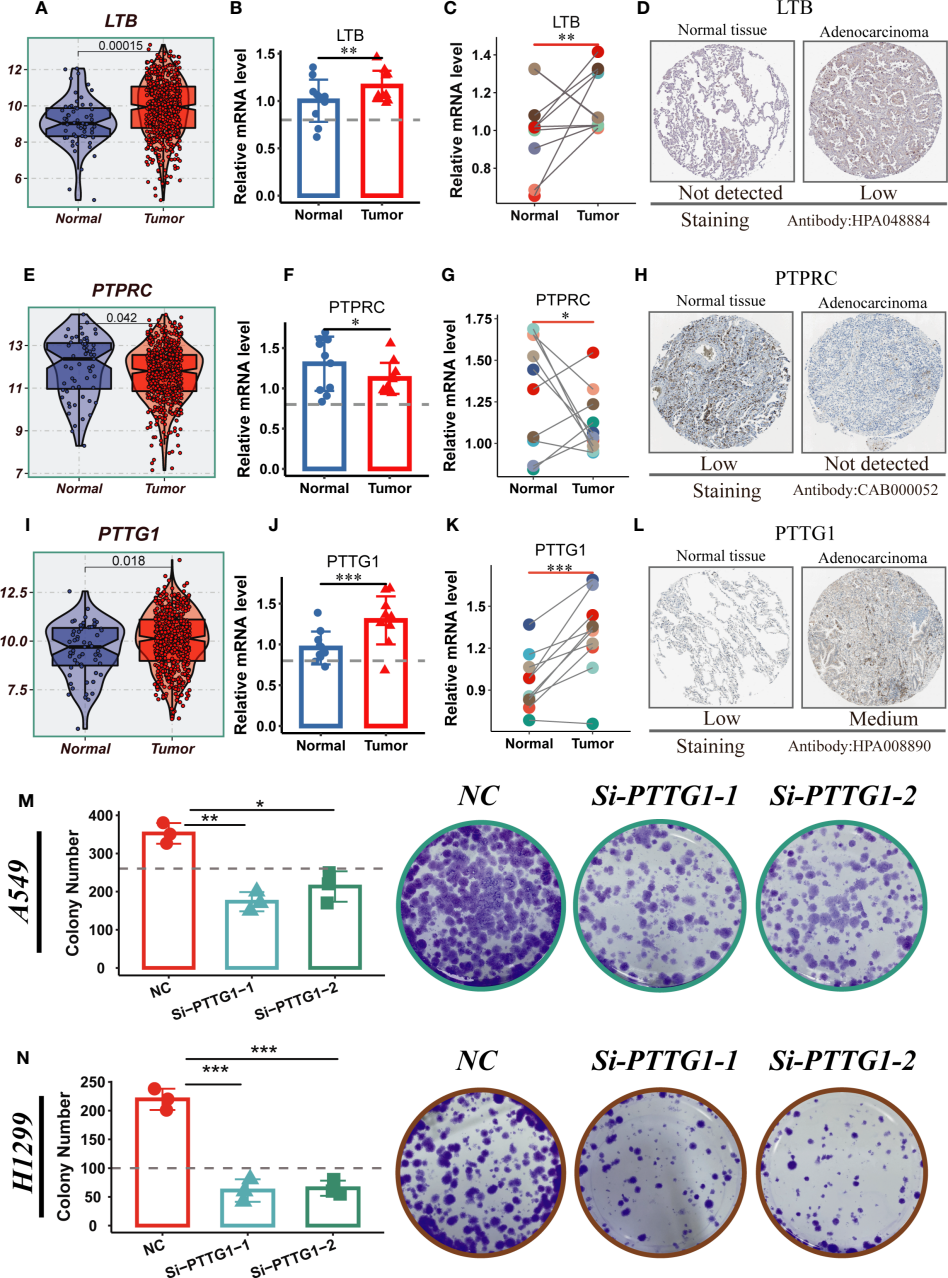
Experimental validation of model gene. **(A)** Boxplots showing the differential expression of LTB between tumor and normal tissues in TCGA-LUAD. **(B–D)** Relative expression of LTB gene in 10 pairs of cancer and paracancer samples, respectively; the HPA database shows the expression of LTB gene in LUAD and normal tissues **(E)** Boxplots showing the differential expression of PTPRC between tumor and normal tissues in TCGA-LUAD. **(F–H)** Relative expression of PTPRC gene in 10 pairs of cancer and paraneoplastic samples, respectively; the HPA database shows the expression of PTPRC gene in LUAD and normal tissues. **(I)** Boxplots showing the differential expression of PTTG1 between tumor and normal tissues in TCGA-LUAD. **(J–L)** Relative expression of PTTG1 gene in 10 pairs of cancer and paraneoplastic samples, respectively; the HPA database shows the expression of PTTG1 gene in LUAD and normal tissues. **(M, N)** Cloning experiments showed that PTTG1 knockdown could significantly reduce the proliferation capacity of LUAD cells. Note: *P < 0.05, **P < 0.01, ***P < 0.001.

LTB, PTPRC, and PTTG1 were significantly different between normal and tumor samples from TCGA-LUAD, whereas the other model genes were not (**Figures 11A, E, I**; **Supplementary Figure S6A**). To validate our findings, we performed qRT-PCR validation using surgically resected tumor and healthy lung tissue. The results showed that LTB and PTTG1 genes were significantly up-regulated in tumor tissues, while PTPRC expression was increased in normal tissues (**Figures 11B, C, F, G, J, K**). Gene validation through immunohistochemistry using the Human Protein Atlas database was performed (**Figures 11D, H, L**). PTTG1 is highly expressed in tumor tissues and has the highest HR value. Furthermore, **Supplementary Figure S4D** indicates that LUAD patients with high PTTG1 expression have poor survival outcomes. In addition, experiments showed that A549 and H1299 LUAD cells exhibited higher PTTG1 expression compared to normal lung cells. Knockdown of PTTG1 resulted in a significant reduction in the number of cell clones in LUAD cell lines (**Figures 11M, N**). These findings strongly suggest that high expression of PTTG1 can promote LUAD cell proliferation.

## 4 Discussion

Immune system plays important role in the development of cancers (45), including Tregs in LC. These specialized immune cells possess immunosuppressive properties and are known to modulate immune responses within the TME. In LC, Tregs can infiltrate the tumor site and suppress the activity of effector T cells, which are responsible for recognizing and eliminating cancer cells. By dampening the immune response, Tregs contribute to the establishment of an immunosuppressive environment that allows tumor cells to evade immune surveillance and promote tumor progression. Inflammatory factors play important functions in disease (46, 47). Tregs exert their suppressive effects through various mechanisms, including the secretion of immunosuppressive cytokines such as interleukin-10 (IL-10) and transforming growth factor-beta (TGF-β). These cytokines inhibit the activation and function of effector T cells, thereby impairing the immune system’s ability to mount an effective anti-tumor response. Additionally, Tregs can directly interact with other immune cells, such as dendritic cells and natural killer cells, further hindering their anti-tumor activities. This intricate network of immune cell interactions orchestrated by Tregs ultimately contributes to immune evasion and tumor immune tolerance in LC. Understanding the role of Tregs in LC is crucial for developing effective immunotherapeutic strategies. Targeting Tregs or modulating their suppressive function holds promise as a potential approach to enhance anti-tumor immune responses and improve the outcomes of LC patients.

Single-cell analysis provides an unparalleled degree of resolution for examining intratumoral heterogeneity, cellular differentiation trajectories, and intercellular communication, thereby presenting promising avenues for applications (48). Through the analysis of cell clustering in scRNA-seq datasets, we have identified genes that are differentially expressed specifically in tumor cells, thereby redirecting the emphasis from comparing tumors to normal tissues, as observed in prior database analyses, towards exploring distinctions among tumor cells themselves (49). In this investigation, we employed scRNA-seq data to discern pivotal marker genes of Tregs. Through the integration of multiple bulk NRA-seq datasets, we devised a prognostic signature comprising seven genes. Subsequently, we computed risk scores to classify patients with LUAD into high-risk and low-risk categories. Comparative analysis of survival curves between the high-risk and low-risk cohorts within the TCGA cohort unveiled enhanced prognoses for individuals belonging to the low-risk group (P<0.05). Analogous survival outcomes were observed across the TCGA-test group, TCGA-train group, and GEO verification group (p<0.05). ROC curve analysis corroborated the elevated accuracy of the devised prognostic signature in appraising the prognostic outlook of LUAD patients at 1, 3, 5, 7, and 10-year intervals, encompassing both the TCGA and GEO cohorts.

Functional enrichment analysis revealed a significant enrichment of TRAS within cell cycle-related pathways, such as mTORC1-signaling and G2M-checkpoint pathways. Inhibition of TBK1 resulted in impaired proliferation, migration, drug resistance, and tumor growth in CRC cells. Furthermore, TBK1 overexpression suppressed the activation of mTORC1-signaling in CRC (50). While concrete evidence regarding the involvement of mTORC1-signaling in the progression of LUAD is lacking, it can be speculated that this pathway also plays a pivotal role in LUAD. The G2M-checkpoint pathway similarly assumes significance in other related malignancies, including pancreatic cancer (51), colorectal cancer (52), breast cancer (53) and more. Manipulating the G2M-checkpoint pathway may also impact the progression of LUAD.

A comprehensive examination of the tumor-infiltrating immune cells can elucidate the mechanisms underlying immune evasion in cancer, thereby offering opportunities to devise innovative therapeutic strategies (54). Through the assessment of immune cell infiltration in high-risk and low-risk groups, this study unveiled a more pronounced abundance of immune cell infiltration in the low-risk group relative to the high-risk group. Previous investigations have established a correlation between the expression levels of immune checkpoint genes and the efficacy of immunotherapy (55). Differential analysis in immune checkpoint gene expression between the high-risk and low-risk groups implied that the low-risk group could derive benefits from treatments targeting additional immune checkpoints. Therapeutic interventions focusing on the TME have emerged as promising modalities in cancer management, given the pivotal role of the TME in modulating tumor progression and response to conventional therapies (56). The TME scoring revealed a higher immune score within the low-risk group compared to the high-risk group, exhibiting a statistically significant disparity. This observation implied that patients belonging to the low-risk group may display heightened susceptibility to immunotherapy.

Leukotriene B4 (LTB) is a key mediator in the cascade of complement, lipid, cytokine and chemokine responses that mediate inflammatory diseases (57). It has been found that LTB can mediate the airway inflammatory response and thus promote the progression of LC, and can be used as a diagnostic marker for LC (58). Pituitary tumor transformation gene-1 (PTTG1), a gene related to DNA repair, was found to be a diagnostic marker for LUAD (59). Protein tyrosine phosphatase receptor type C (PTPRC) may be a potential prognostic marker for LUAD, and it may affect the function of γδT cells and other immune cells by being involved in the regulation of TME immune status (60). In our study, we verified that LTB and PTTG1 were highly expressed in tumor tissues and PTPRC was highly expressed in normal tissues using clinical surgical resection samples, but further mechanistic experiments are needed to verify the specific functions of these model genes.

The constructed TRAS in this study facilitates the prognostic prediction of LUAD patients and unveils potential avenues for the implementation of immunotherapy. Nevertheless, additional experimental investigations are imperative to authenticate these discoveries. Furthermore, the migratory capacity of tumor cells is closely associated with adverse prognosis and tumor recurrence (61), while drug resistance, metabolic reprogramming, and epigenetic alterations also play crucial roles in the progression of tumor patients’ prognosis (62, 63). Our study lacks analysis in this area. In future investigations, we will focus on exploring the impact mechanisms of Tregs in these aspects within lung cancer.

## Data availability statement

The original contributions presented in the study are included in the article/**Supplementary Material**. Further inquiries can be directed to the corresponding authors.

## Ethics statement

The studies involving human participants were reviewed and approved by The Ethics Committee of the First Affiliated Hospital of Nanjing Medical University has approved all human experiments conducted in this study (Approval No. 2019-SR-156). The patients/participants provided their written informed consent to participate in this study.

## Author contributions

The study was conceived and designed by PZ, XZ, and ZG. Data collection was conducted by YC. PZ and XZ performed the statistical analysis. The first draft of the manuscript was written by PZ and WW. The final approval of the submitted version was given by SL. All authors contributed to the manuscript and approved the submitted version. All authors contributed to the article and approved the submitted version.
