# Supplementary figures and images for: Revealing the role of regulatory T cells in the tumor microenvironment of lung adenocarcinoma: a novel prognostic and immunotherapeutic signature
Source: Front Immunol. 2023 Aug 21;14:1244144. doi: 10.3389/fimmu.2023.1244144 (PMC10476870; doi:10.3389/fimmu.2023.1244144)

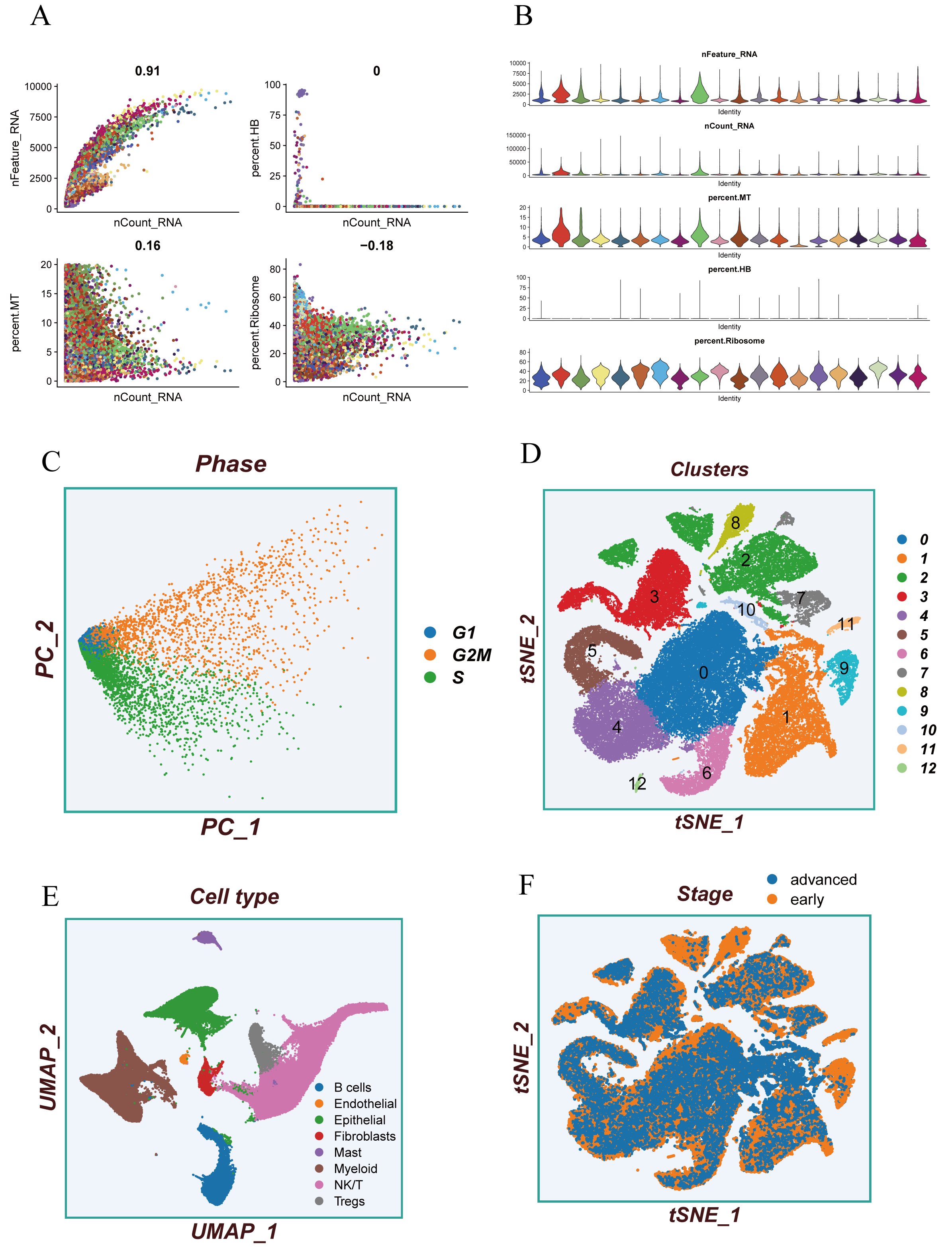

Supplement: Supplementary file 1 [file Image_1.tif]

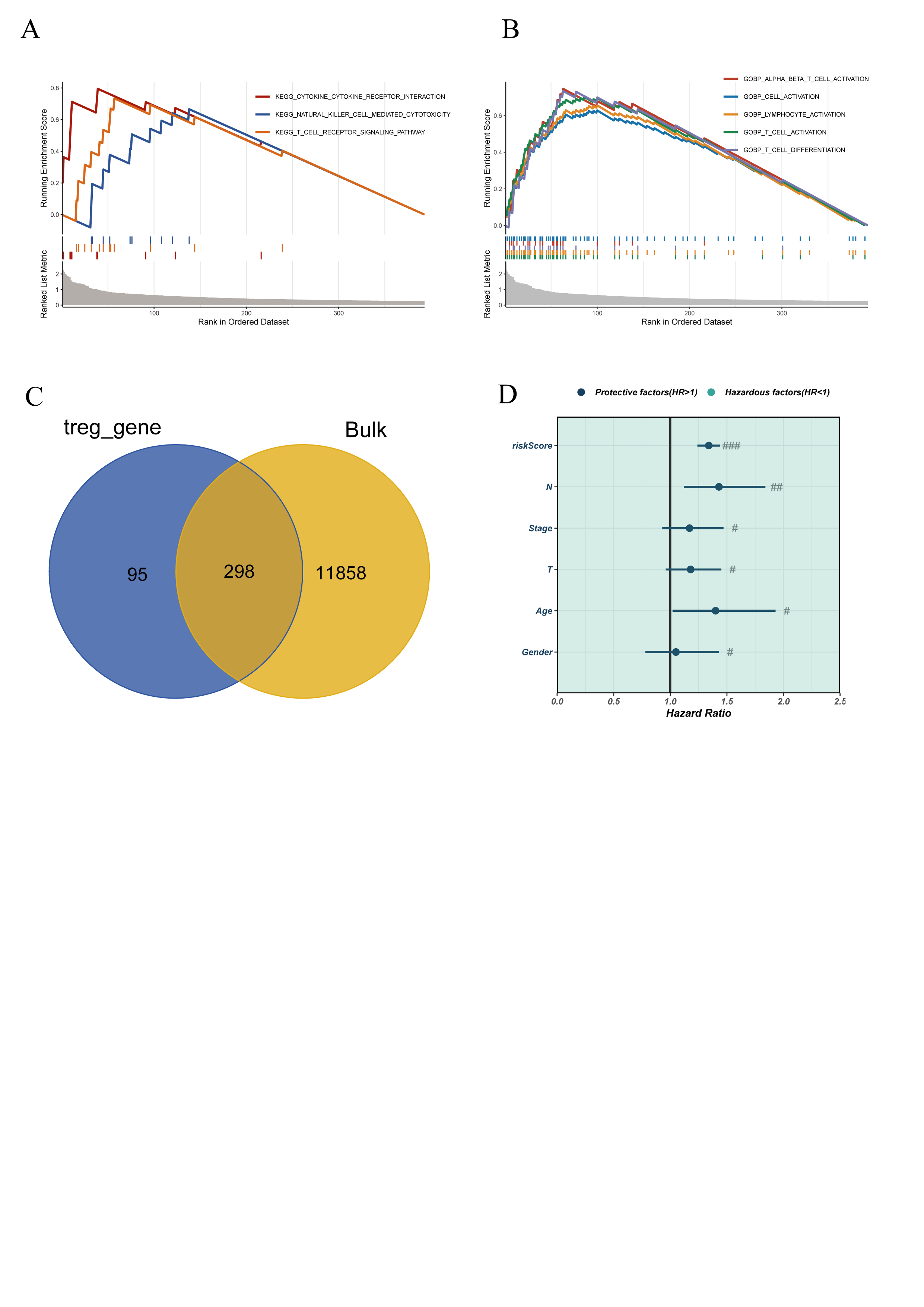

Supplement: Supplementary file 2 [file Image_2.tif]

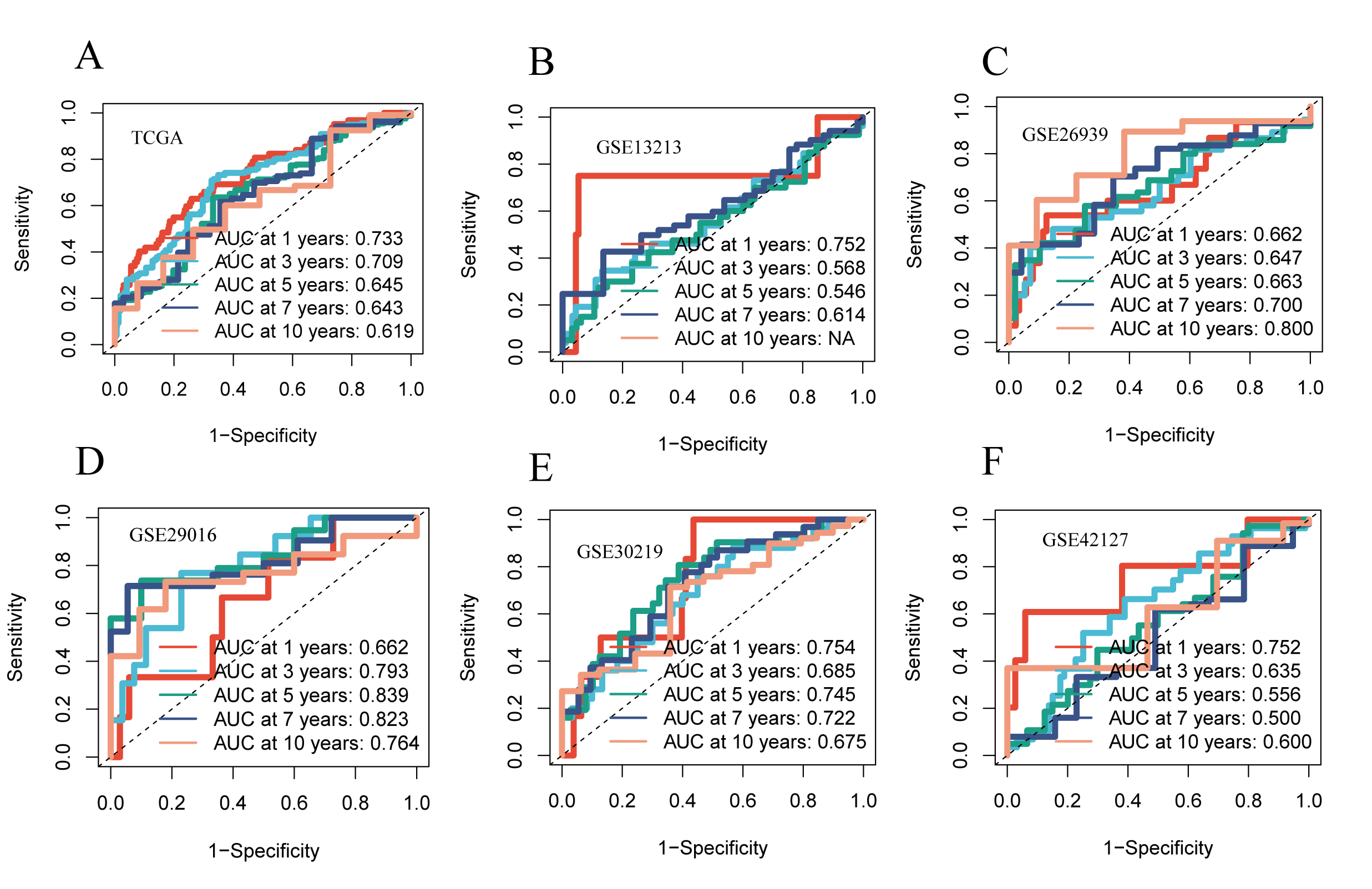

Supplement: Supplementary file 3 [file Image_3.tif]

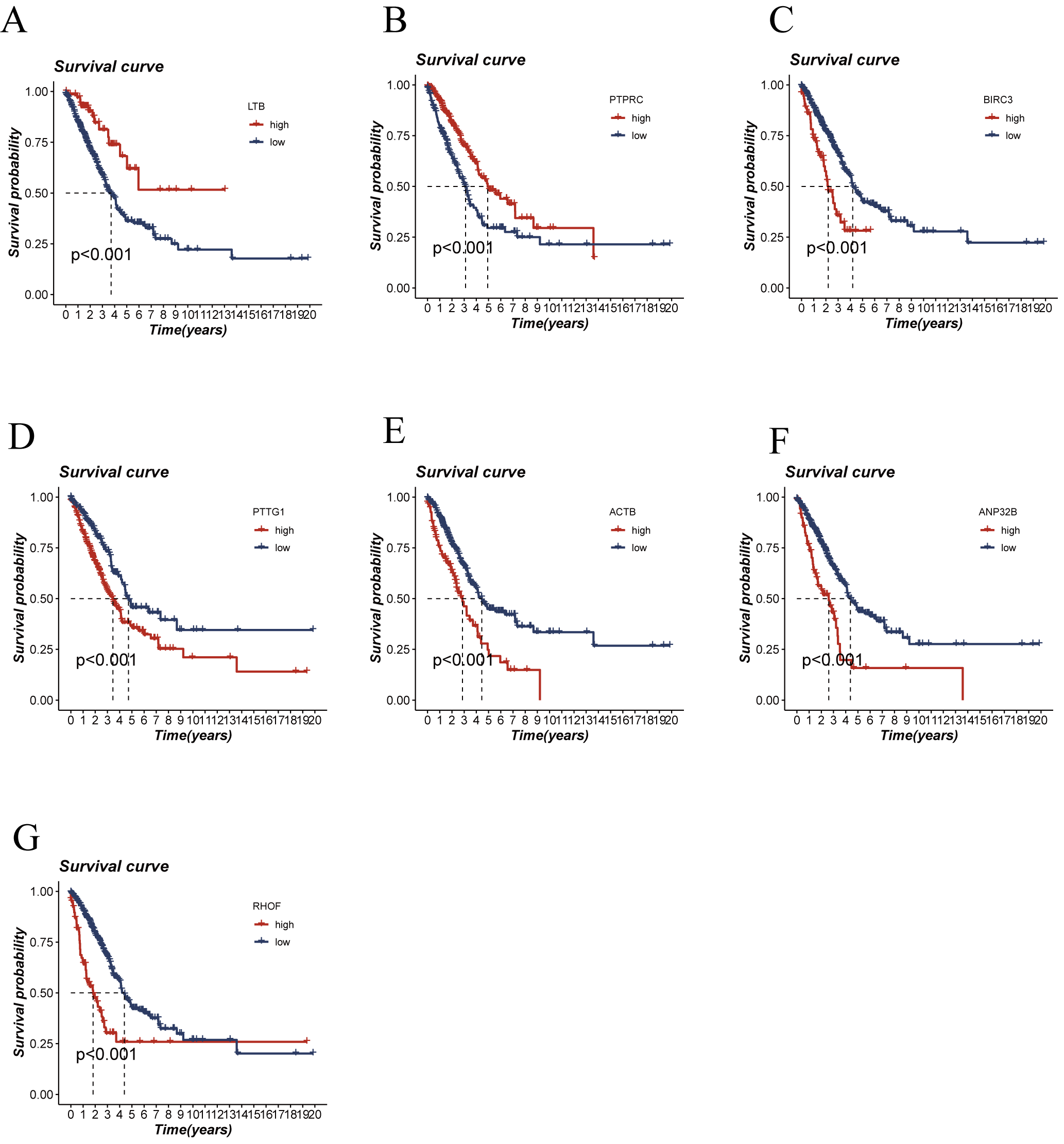

Supplement: Supplementary file 4 [file Image_4.tif]

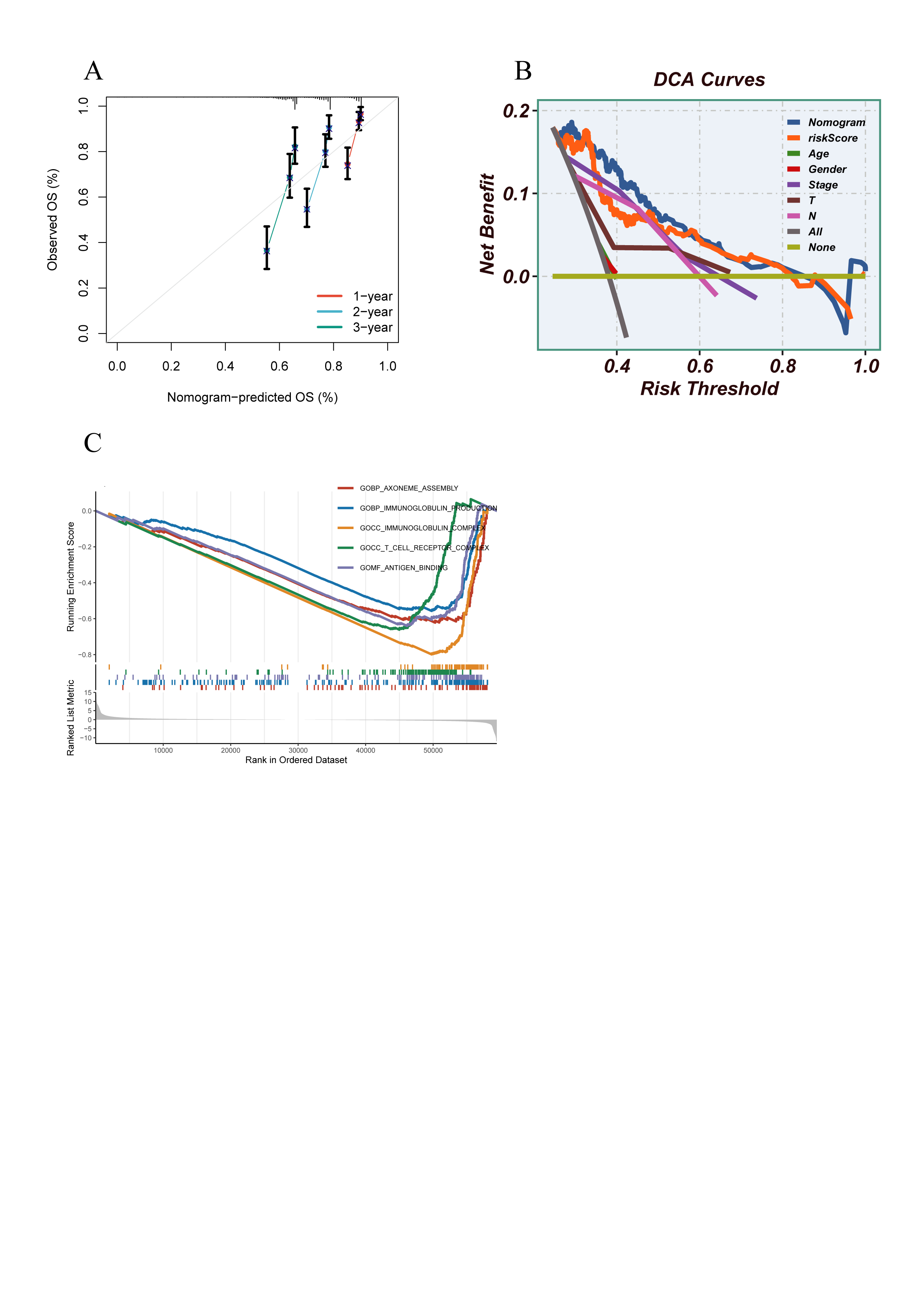

Supplement: Supplementary file 5 [file Image_5.tif]

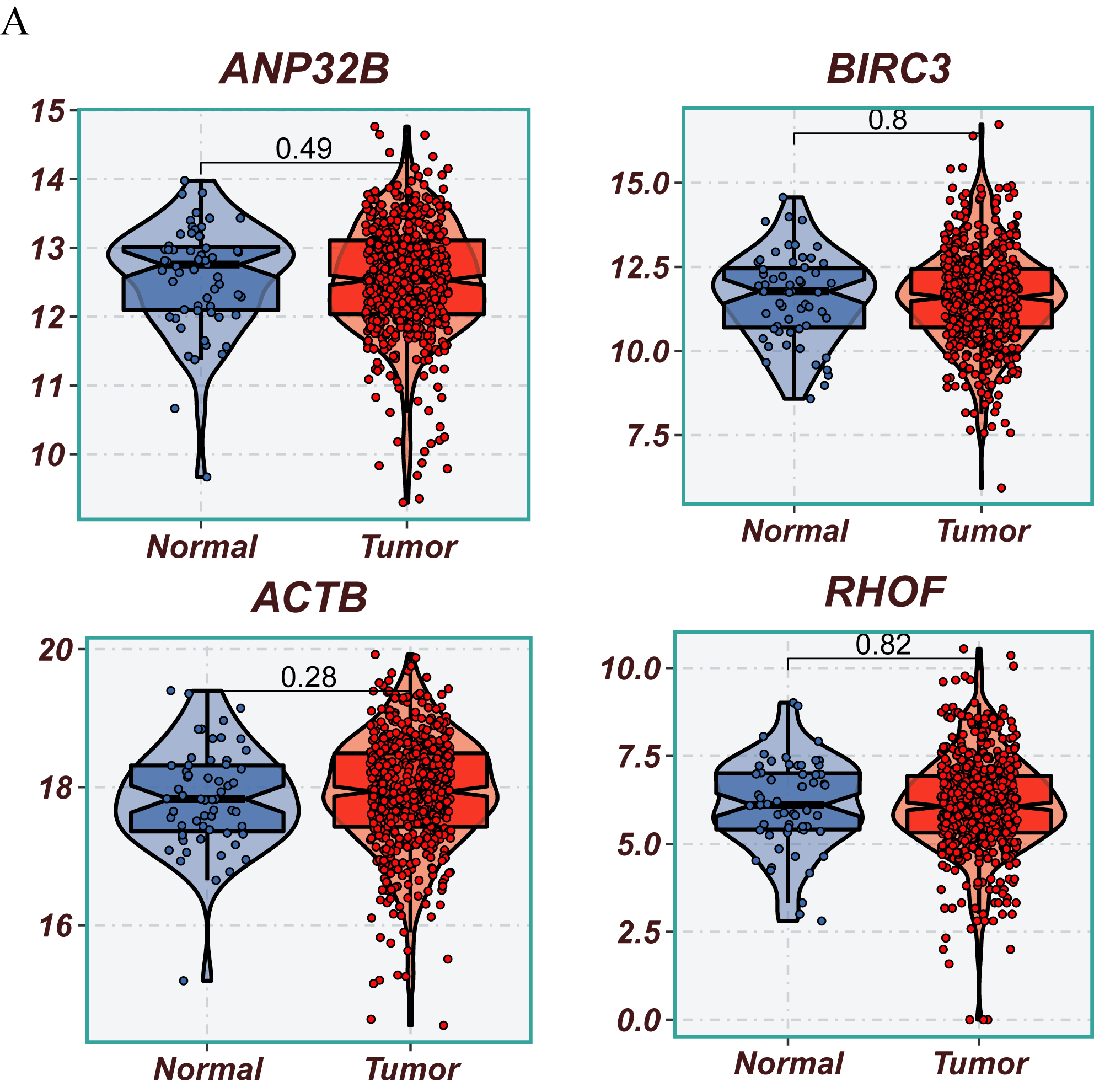

Supplement: Supplementary file 6 [file Image_6.tif]
